# Effects of different water management options and fertilizer supply on photosynthesis, fluorescence parameters and water use efficiency of *Prunella vulgaris* seedlings

**DOI:** 10.1186/s40659-016-0069-4

**Published:** 2016-02-24

**Authors:** Yuhang Chen, Li Liu, Qiaosheng Guo, Zaibiao Zhu, Lixia Zhang

**Affiliations:** Institute of Chinese Medicinal Materials, Nanjing Agricultural University, Nanjing, 210095 People’s Republic of China; College of Pharmaceutical Sciences, Chengdu Medical College, Chengdu, 610083 People’s Republic of China

**Keywords:** *Prunella vulgaris* L, N, P and K fertilizer, Drought, Photosynthesis, Water use efficiency

## Abstract

**Background:**

*Prunella vulgaris* L. is a medical plant cultivated in sloping, sun-shaded areas in China. Recently, owing to air-environmental stress, especially drought stress strongly inhibits plant growth and development, the appropriate fertilizer supply can alleviate these effects. However, these is little information about their effects on *P. vulgaris* growing in arid and semi-arid areas with limited water and fertilizer supply.

**Results:**

In this study, water stress decreased the photosynthetic pigment contents, inhibited photosynthetic efficiency, induced photodamage in photosystem 2 (PS2), and decreased leaf instantaneous WUE (WUEi). The decreased net photosynthetic rate (Pn) under medium drought stress compared with the control might result from stomatal limitations. However, fertilizer supply improved photosynthetic capacity by increasing the photosynthetic pigment contents and enhancing photosynthetic efficiency under water deficit. Moreover, medium fertilization also increased WUEi under the two water conditions, but fertilizer supply did little to alleviate the PS2 photodamage caused by drought stress. Hence, drought stress was the primary limitation in the photosynthetic process of *P. vulgaris* seedlings, while the photosynthetic characteristics of the seedlings exhibited positive responses to fertilizer supply.

**Conclusions:**

Appropriate fertilizer supply is recommended to improve photosynthetic efficiency, enhance WUEi and alleviate photodamage under drought stress.

## Background

*Prunella vulgaris* L. (Labiatae), also known as “self-heal,” is a popular ingredient in the preparation of traditional Chinese medicine [1]. The dried spica of *P. vulgaris*, i.e., Prunellae Spica, is traditionally used as herbal medicine to alleviate fever, reduce sore throats, and accelerate wound healing [2, 3]. The spicae have been shown to possess antioxidant, anticancer, anti-lipid peroxidation, anti-inflammatory, anti-hyperglycemia and hepatoprotective activities [2]. In traditional Chinese medicine, the air-dried plants are widely used to prepare functional tea, and the leaves are also consumed as medicinal vegetables [4, 5].

Due to its medicinal and industrial importance, the demand for *P. vulgaris* has increased steadily in recent years [6]. The wild population of *P. vulgaris* cannot meet this growing need, and therefore it was proposed in the 1990s that *P. vulgaris* be cultivated to allow for more efficient resource utilization in China [1]. *P. vulgaris* was originally classified as a moderate shade species, especially during the seedling stage. Strong irradiation at midday usually induces severe photoinhibition and photo-oxidative damage of the photosynthetic apparatus of *P. vulgaris* leaves [7]. A number of environmental stresses, including drought and malnutrition, may increase *P. vulgaris* plant sensitivity to photoinhibition and photodamage, inducing cellular damage and thus decreasing their productivity [1, 4, 5].

*Prunella vulgaris* plants require moderate levels of nutrients and are sensitive to drought [1, 6]. The growth and yield of *P. vulgaris* are restricted by water and nutrient deficiencies because most *P. vulgaris* plantations are located in mountainous areas in China. Soil water deficit in the dry season is one of the most important limitations to photosynthesis and consequently, *P. vulgaris* productivity [1, 6]. However, there are numerous well-documented photosynthetic responses of plants to N, P, and K fertilization, which include significant and positive correlations between photosynthetic capacity and leaf N, P, and K content, suggesting that a large proportion of these elements is used for the synthesis of various components in the photosynthetic apparatus [6, 8]. Furthermore, fertilization (e.g., N, P, K) frequently increases cell wall rigidity and osmotic adjustment [6, 8]. Increased fertilization might improve the photosynthetic capacity or stomatal control under water and nutrient deficit conditions.

Under drought stress, disturbances in photosynthesis at the molecular level are connected with low electron transport through photosystem 2 (PS2) and/or with structural injuries of PS2 and the light-harvesting complexes [9, 10]. Restricted CO_2_ may lead to increased susceptibility to photodamage (due to stomatal closure) and, subsequently, to photoinhibition [11]. Photoinhibition is characterized by parallel decreases in Pn and quantum yield of PS2 (Ф PS2) and is accompanied by a decline in the maximum quantum yield of photosynthesis (Fv/Fm) associated with loss of PS2 activity [11, 12] and an increase in minimal Chl fluorescence (F0) [13]. Chl fluorescence is a useful tool for quantifying the effect of abiotic stress on photosynthesis [12]. Fertilizer could increase Fv/Fm and the effective quantum yield of photochemical energy conservation in PS2 (Fv′/Fm′) [14]. The Chl content and photosynthetic rates might also be enhanced through the supply of N, P, and K [6, 8]. These authors suggested that fertilization might alleviate photoinhibition and photodamage caused by drought stress.

The interactive effects of nutrition and water availability on *P. vulgaris* growth and the production of secondary metabolites have been well documented [1, 6, 7]. However, the physiological and biochemical characteristics of *P. vulgaris* plants under drought and nutrient-limited conditions have been less thoroughly studied. The objectives of this study were to (1) determine the photosynthetic adaptation of *P. vulgaris* seedlings to various water supply and fertilization conditions, and (2) determine whether fertilization could improve the photosynthetic capacity of seedlings under dry conditions.

## Results

### Diurnal variation of environmental factors

**Fig. 1:**
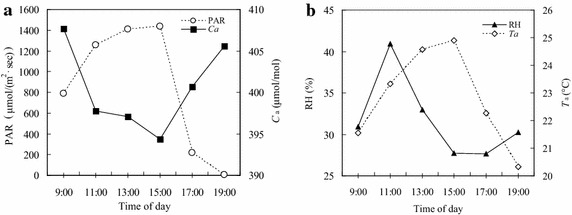
Diurnal variations of environmental photosynthetically active radiation (PAR) and ambient CO_2_ concentration (Ca) (**a**); air temperature (Ta) and air relative humidity (RH) (**b**) from 09:00 to 19:00 on the measuring day

On the measurement day, the PAR increased steeply from 09:00 to 11:00, remained at high levels until 15:00, and then decreased sharply (Fig. 1a). Under the impacts of PAR diurnal variation, Ca (Fig. 1a) and RH (Fig. 1b) were at high levels in the early morning, followed by a sharp decrease, remaining at relatively low levels during the midday period, and then began to increase from 15:00. In contrast, Ta exhibited a diurnal trend similar to PAR (Fig. 1b).

**The contents of the photosynthetic pigments** exhibited significant (*P* < 0.05) responses to water stress and fertilization supply, and the interaction between water and fertilization affected Chl*a* and *b*, Chl*a* + *b* and Car (Table 1). Water stress decreased the contents of Chl*a* and *b*, Chl*a* + *b* and Car. On the other hand, significantly higher values were measured in Fl compared with the other fertilization treatments (F0 and F2) under well-watered and drought stress conditions.

**Table 1:** Chlorophyll (Chl) a and b contents, Chl *a* + *b* content, carotenoid (Car) content of *P. vulgaris* seedlings under different water (W) and fertilization (F) supply regimes

Treatment	Chl*a* [g kg^−1^ (FM)]	Chl*b* [g kg^−1^ (FM)]	Chl*a* + *b* [g kg^−1^ (FM)]	Car [g kg^−1^ (FM)]
W1/F0	0.40 ± 0.06 d	0.16 ± 0.03 d	0.57 ± 0.10 d	0.10 ± 0.01 d
W1/F1	0.86 ± 0.09 b	0.34 ± 0.04 b	1.19 ± 0.13 b	0.20 ± 0.02 b
W1/F2	0.61 ± 0.10 c	0.25 ± 0.04 c	0.86 ± 0.14 c	0.15 ± 0.02 c
W2/F0	0.42 ± 0.01 d	0.17 ± 0.00 d	0.58 ± 0.02 d	0.12 ± 0.00 d
W2/F1	1.03 ± 0.10 a	0.41 ± 0.03 a	1.45 ± 0.13 a	0.24 ± 0.02 a
W2/F2	1.00 ± 0.04 a	0.40 ± 0.02 a	1.40 ± 0.06 a	0.21 ± 0.01 b
Water (W)	43.68**	43.83**	44.24**	28.78**
Fertilization (F)	120.30**	116.13**	120.52**	89.46**
W × F	14.24**	12.91**	14.01**	3.51

W1 and W2 correspond to soil water contents between 45–50 and 70–75 % of the field water capacity, respectively; F0: no fertilization, F1: 0.12 g N + 0.2 g P_2_O_5_ + 0.1 g K_2_O kg^−1^ soil, F2: 0.24 g N + 0.4 g P_2_O_5_ + 0.2 g K_2_O kg^−1^ soil. Different letters indicate significant differences between treatments at *P* < 0.05 (ANOVA)

Mean ± SD, fresh mass (FM). n = 6, *P < 0.05, **P < 0.01

### Water supply and fertilization effects on the diurnal variation of photosynthetic parameters

#### Diurnal variation of Tr

**Fig. 2:**
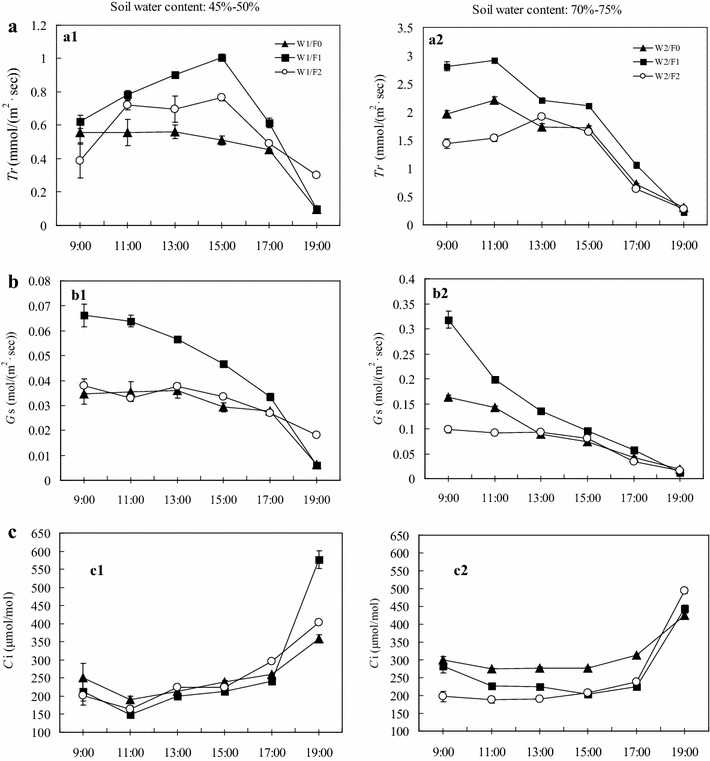
Diurnal variations in leaf transpiration (Tr) (**a**), stomatal conductance (Gs) (**b**), intercellular CO_2_ concentration (Ci) (**c**) of* P. vulgaris* exposed to two soil water (W) conditions and three levels of fertilizer (F), n = 6–8. F0: no fertilization, F1: 0.12 g N + 0.2 g P_2_O_5_ + 0.1 g K_2_O kg^−1^ soil, F2: 0.24 g N + 0.4 g P_2_O_5_ + 0.2 g K_2_O kg^−1^ soil

Transpirational water loss was compensated for at dusk every day. Hence, based on the diurnal variation of the main environmental factors affecting transpiration (Fig. 1), under drought stress, leaf Tr was maintained at a relatively high level from 09:00 to 15:00, followed by a significant and continuous decrease, reaching the lowest value of the day at 19:00 (Fig. 2a1). Under well-watered conditions, leaf Tr was maintained at a relatively high level from 09:00 to 15:00, followed by a significant and continuous decrease, reaching the lowest value of the day at 19:00 (Fig. 2a2). Irrespective of fertilizer supply, Tr decreased with decreasing soil water availability at each measurement point during the day. For a particular water content, fertilizer supply significantly affected Tr (Table 2).

**Fig. 3:**
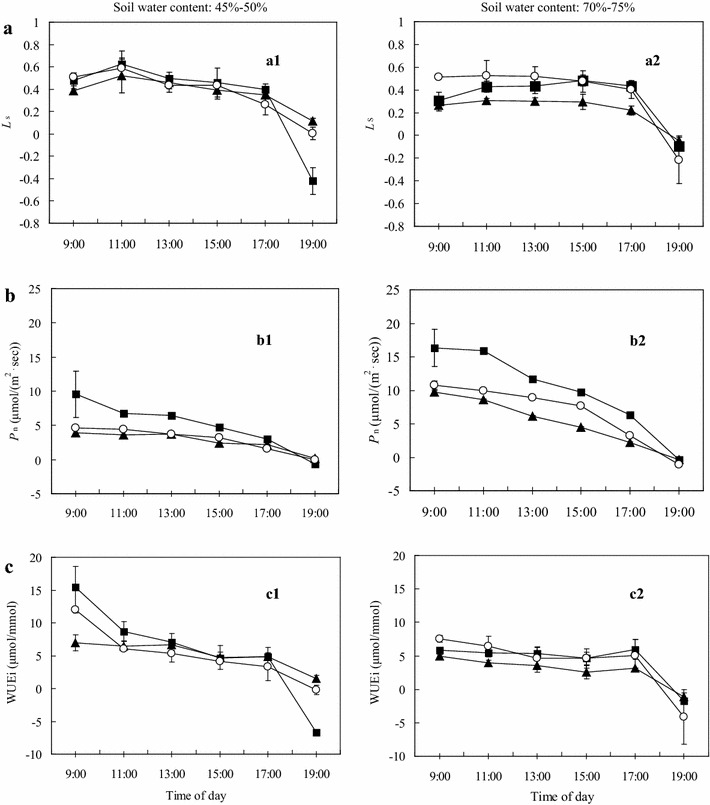
Stomatal limitation values (Ls) (**a**), net photosynthetic rate (Pn) (**b**), and leaf instantaneous water use efficiency (WUEi) (**c**) of *P. vulgaris* exposed to two soil water (W) conditions and three levels of fertilizer (F), n = 6–8. F0: no fertilization, F1: 0.12 g N + 0.2 g P_2_O_5_ + 0.1 g K_2_O kg^−1^ soil, F2: 0.24 g N + 0.4 g P_2_O_5_ + 0.2 g K_2_O kg^−1^ soil

**Table 2:** Statistical tests of the effects of three fertilization levels on Tr, Gs, Ci, Ls, Pn, and WUEi of *P. vulgaris* seedlings at various measuring times and soil water availabilities

Treatment %	09:00	11:00	13:00	15:00	17:00	19:00
Effects of fertilization supply on Tr						
45–50	–	–	*	*	*	*
70–75	*	*	–	*	–	–
Effects of fertilization supply on Gs						
45–50 %	*	*	*	*	–	*
70–75 %	*	*	–	–	*	*
Effects of fertilization supply on Ci						
45–50	*	–	–	*	*	*
70–75	*	*	*	*	*	–
Effects of fertilization supply on Ls						
45–50	*	–	–	*	*	*
70–75	*	*	*	*	*	–
Effects of fertilization supply on Pn						
45–50	*	*	*	*	*	*
70–75	*	*	*	*	*	–
Effects of fertilization supply on WUEi						
45–50	*	*	*	–	–	*
70–75	*	*	–	*	*	–

The seedlings were exposed to two soil water conditions (non-limiting soil water content and medium drought, corresponding to soil water contents between 70–75 % and 45–50 % of the field water capacity, respectively) and three levels of fertilization (F0: no fertilizer, F1: 0.12 g N + 0.2 g P_2_O_5_ + 0.1 g K_2_O kg^−1^ soil, F2: 0.24 g N + 0.4 g P_2_O_5_ + 0.2 g K_2_O kg^−1^ soil; n = 6 for each treatment)

* Significant differences between fertilization treatments at a specific time of day for a particular soil water content (ANOVA, *P* < 0.05), – indicates no significant difference

#### Diurnal variation of Gs

Stomata are the main portals for carbon dioxide (CO_2_) and vapor water exchange between plant leaves and the atmosphere; thus, Gs directly controls photosynthesis and transpiration. The diurnal variation of Gs under all water and fertilization treatments showed similar trends (Fig. 2b). At 09:00, Gs was maintained at relatively high levels due to compensation for transpirational water loss at dusk the day before. Then, Gs decreased significantly until dusk. Irrespective of the fertilization level, Gs decreased with decreasing soil water content. At comparable soil water contents, the Gs of plants receiving the moderate level of fertilization was consistently higher than that of the high fertilization plants and those plants that did not receive fertilizer. In the afternoon, the effect of fertilizer supply on Gs under non-limiting water conditions and medium drought was statistically significant (Table 2).

#### Diurnal variation of Ci

In general, Ci is dependent on Gs and the ability of the mesophyll cells to assimilate intracellular CO_2_. At 09:00, Ci was high (Fig. 2c), consistent with high Ca, and associated with low PAR and high Gs. During the period from mid-morning to early evening (09:00–17:00), Ci reached a constant low value due to high PAR, depletion of Ca in the plant canopy, and high Gs, which facilitated Ca depletion (Fig. 1a), followed by a return to the early morning levels as the light and Gs decreased. Increasing soil water content did not change the trend in diurnal Ci variation, leading to only a weak increase in Ci under both moderate and high fertilizer supply in the late afternoon (Fig. 2c1, c2). Compared with the drought stress treatments (Fig. 2c1), the Ci of well-watered treatments (Fig. 2c2) was higher during the middle of the day but significantly lower in the late afternoon under moderate fertilizer supply (Table 2).

#### Diurnal variation of Ls

The diurnal changes in Ls displayed a similar pattern under the well-watered and drought conditions (Fig. 3a). Ls increased with an increase in water stress, and moderate fertilization also increased Ls under drought stress and well-watered conditions. Compared with the well-watered treatment (Fig. 3a2), the high fertilization treatment and the control (Fig. 3a1) reduced stomatal limitations during the middle of the day and in the early morning in the drought stress treatments. This suggests that moderate fertilizer supply modified the stomatal limitation of CO_2_ diffusion for most of the day, especially in the seedlings grown under drought stress conditions (Table 2).

#### Diurnal variation of Pn

Diurnal variations of Pn are shown in Fig. 3b. Overall, the pattern of Pn mirrored that of Gs. Pn and Gs maxima occurred at 09:00 as PAR and Gs increased, even though Ci was reduced. Subsequently, Pn steadily declined in all water and fertilization treatments until a minimum value was reached in the early evening (19:00), similar to Gs. Hence, Gs was the main factor limiting the photosynthesis of mesophyll cells at this time of day. At 19:00, Pn was at the lowest level of the day due to low PAR and Gs, despite the high Ci.

Water and fertilization did not affect the trend in the diurnal variation of Pn (Fig. 3b). Independent of soil N + P + K, increasing soil water availability increased Pn. Under all soil water contents, the Pn of the moderate fertilization treatment was higher than that of the high fertilization treatment and the control. The fertilization effects under non-limiting water conditions and medium drought were significantly different at each measurement point (Table 2).

#### Diurnal variation of WUEi

Similar to the trend in the diurnal variation of Pn, WUEi (Fig. 3c) reached a maximum level at 09:00 due to high Pn and PAR. Throughout the remainder of the day, WUEi declined as Ta increased and RH (Fig. 1b) and Pn decreased, while Tr remained at relatively high levels (Fig. 2a). A decrease in the soil water content led to an increase in the WUEi when the plants were grown under moderate fertilizer levels, compared with a decrease in the WUEi when the plants were grown under high fertilization (Fig. 3c1). A comparison of the control seedlings and those exposed to high fertilization under drought conditions indicated that an increase in fertilization enhanced the WUEi of *P. vulgaris* seedlings (Table 2).

#### Chl fluorescence

Fertilization significantly affected the Chl fluorescence parameters (Table 3). In addition, water stress significantly affected the following Chl fluorescence parameters: ФPS2, qP, qN, Fv′/Fm′ and ETR, although qP and ETR showed significant responses to the interaction of water and fertilization. Water stress decreased Fv/Fm, ФPS2 and ETR, while Fl increased these parameters under drought and well-watered conditions. qP was slightly decreased with an increase in drought stress, whereas qN was increased. Fv′/Fm′ decreased under drought stress but responded somewhat positively to Fl.

**Table 3:** The maximum quantum yield of photosystem 2 (PS2) photochemistry (Fv/Fm), effective quantum yield of PS2 (ФPS2), photochemical quenching (qP), non-photochemical quenching (qN), effective quantum yield of photochemical energy conservation in PS2 (Fv′/Fm′) and electron transport rate (ETR) of *P. vulgaris* seedlings under different water (W) and fertilization (F) regimes

Treatment	Fv/Fm	ФPS2	QP	QN	Fv′/Fm′	ETR
W1/F0	0.80 ± 0.03 b	0.18 ± 0.02 c	0.46 ± 0.06 c	1.82 ± 0.06 ab	0.39 ± 0.01 e	90.45 ± 4.26 c
W1/F1	0.84 ± 0.01 a	0.26 ± 0.01 b	0.62 ± 0.02 ab	1.91 ± 0.04 a	0.43 ± 0.01 cd	131.12 ± 3.74 b
W1/F2	0.82 ± 0.01 a	0.24 ± 0.04 b	0.58 ± 0.05 b	1.91 ± 0.14 a	0.41 ± 0.03 de	114.19 ± 20.03 b
W2/F0	0.80 ± 0.01 b	0.26 ± 0.01 b	0.58 ± 0.01 b	1.64 ± 0.03 c	0.45 ± 0.02 bc	92.56 ± 9.95 c
W2/F1	0.84 ± 0.01 a	0.32 ± 0.01 a	0.68 ± 0.00 a	1.75 ± 0.03 bc	0.48 ± 0.01 a	168.02 ± 7.14 a
W2/F2	0.83 ± 0.01 a	0.30 ± 0.05 a	0.56 ± 0.17 b	1.69 ± 0.07 c	0.45 ± 0.02 ab	150.70 ± 22.53 a
Water (W)	0.69	56.76**	19.77**	69.68**	110.67**	38.14**
Fertilization (F)	29.97**	25.42**	27.43**	6.87**	14.45**	72.94**
W × F	1.33	0.79	4.56*	0.41	0.58	8.00**

W1 and W2 correspond to soil water contents between 45–50 and 70–75 % of the field water capacity, respectively; F0: no fertilization, F1: 0.12 g N + 0.2 g P_2_O_5_ + 0.1 g K_2_O kg^−1^ soil, F2: 0.24 g N + 0.4 g P_2_O_5_ + 0.2 g K_2_O kg^−1^ soil. Different letters indicate significant differences between treatments at *P* < 0.05 (ANOVA)

Mean ± SD, n = 5, *P < 0.05, **P < 0.01

## Discussion

As expected, the photosynthetic characteristics exhibited strong responses to drought stress and fertilization, which is in agreement with many previous studies [10, 15, 16]. Nevertheless, we found that drought stress seemed to play the primary limiting role in photosynthetic capacity, which was improved by fertilization, but the decreasing tendency could not be altered.

Lower photosynthetic performance of *P. vulgaris* seedlings may be associated with decreasing Chl and Car contents under water stress. However, the highest photosynthetic pigment contents were measured in the moderate fertilization treatment under water stress, which implied that moderate fertilization could alleviate the damages caused by water stress and improve photosynthetic performance under water deficit. Similar results have been observed in *Sophora davidii* seedlings [10] and *Doritaenopsis* seedlings [17].

Both drought stress and fertilization slightly affected the diurnal fluctuation patterns of *P. vulgaris* seedlings, which are strongly related to the biological rhythm of the plant [10, 16]. Most studies have reported positive effects of fertilizer supply on plant photosynthesis and WUE [16, 18], although negative effects on WUE in response to fertilizer supply have been observed in some experiments [18, 19]. In this study, an increase in soil nutrient availability did not alter the trend in the diurnal variations of the photosynthetic parameters measured in response to soil water availability. However, compared with the plants grown under drought stress conditions, fertilization generally increased Gs and Ci, diminished Ls, and enhanced Pn. Fertilization enhanced WUEi under non-limiting water and medium drought conditions, which was in agreement with the results of previous study [20] and the general theory that the supply of a limited resource can enhance the use efficiency of other resources. Generally, an increase in soil water availability is more effective than an increase in nutrient availability in improving the growth of *P. vulgaris* seedlings. Although differences between pot studies and field experimental conditions often limit the practical application of pot experimental results, this study provides useful data for the management of the early phase of *P. vulgaris* seedlings.

The efficiency and stability of PS2 have been widely monitored through the measurement of Fv/Fm in dark-adapted leaves [14]. In our study, water stress increased Fv/Fm, which implies that drought stress had a greater effect on the energy cycling (Fm) between the reaction center (RC) and the Chl pool compared with the energy absorption rate of the leaves (F0) [21]. Moderate fertilization led to apparent modifications of F0, Fm and Fv/Fm, which might alleviate photoinhibition or other types of PS2 injuries caused by drought stress. This is consistent with the findings in *Sophora davidii* seedlings [10].

For monitoring the efficiency of photochemical processes in PS2 in a light-adapted state, Ф PS2 and Fv′/Fm′ are usually used [22]. In our measurements, both parameters exhibited negative responses to drought stress and positive responses to moderate fertilization under well-watered and drought stress conditions, suggesting that water stress decreased the efficiency of excitation energy capture of open PS2 RCs, whereas fertilization supply might improve this under medium drought stress. Similar results were also observed in *Sophora davidii* seedlings responding to drought stress [10]. However, excess fertilizer supply might strongly aggravate the damage caused by drought stress.

Two basic parameters describe the quenching of maximum variable Chl fluorescence yield during the irradiation induction period: qP and qN. In our study, decreased qP under drought stress suggested that drought stress might damage PS2 RCs, resulting in their closure. Higher qN under drought stress indicated that plants efficiently dissipated the energy trapped in PS2 in the form of heat. This is the photoprotective mechanism under stress [23].

In the present study, the ETR of PS2 decreased under the drought condition, which indicated that the proportion of open reaction centers of PS2 and CO_2_ fixation were reduced. The results indicated that photosynthetic electron transport ability was reduced and the dark reaction was blocked, which decreased the photosynthetic rate [24].

## Conclusion

In conclusion, drought stress not only decreases the contents of the photosynthetic pigments, the photosynthetic capacity, and the WUEi but also affects the efficiency of PS2 of *P. vulgaris* seedlings. However, the photosynthetic pigments and gas exchange responded positively to fertilization. In addition, fertilization alleviated the degree of photo-inhibition and the injury caused by drought stress by slightly improving Fv/Fm and increasing Fv′/Fm′. Thus, appropriate fertilization is recommended for *P. vulgaris* seedlings to improve photosynthesis inhibited by drought stress and to facilitate seedling establishment under water deficit.

## Methods

### Plants and their growth

Seeds of *P. vulgaris* were collected in July 2009 in Queshan County, Henan Province, P.R. China. Apparently healthy seeds were air-dried and then stored at ambient laboratory temperature until the experimental pre-treatment was initiated in October 2009. Surface soil from an experimental field at Nanjing Agricultural University was used as the growth substrate. The collected soil was combined and thoroughly mixed. Soil (4.0 kg) was placed in each 4.5 L plastic pot. The organic matter content of the soil was 21.32 g kg^−1^; available N was 34.65 g kg^−1^; available P was 12.07 g kg^−1^; and available K was 16.34 g kg^−1^. The field capacity of the soil was 25 %.

Before sowing, *P. vulgaris* seeds were soaked in 2.5 % sodium hypochlorite solution for 1 h. Twenty seeds of approximately the same size were sown in each pot on 10 October 2009. All pots were moved into a rain shelter located at the Institute of Chinese Medicinal Materials, Nanjing Agricultural University, Nanjing, Jiangsu Province, P.R. China. All pots were well watered to ensure germination. After one month, the seedlings were thinned to four uniform plants per pot.

### Experimental design

The experiment was arranged using a randomized design consisting of two water regimens [70–75 and 45–50 % of field water capacity (FWC)] and three fertilizer treatments (N0P0K0 = no fertilization (control), N1P1K1 = 0.12 g N + 0.2 g P_2_O_5_ + 0.1 g K_2_O kg^−1^ soil, N2P2K2 = 0.24 g N + 0.4 g P_2_O_5_ + 0.2 g K_2_O kg^−1^ soil). The N, P_2_O_5_, and K_2_O were applied as urea, superphosphate and potassium sulfate, respectively. One third of the N and all of the P and K were applied basally. The remaining N fertilizer was applied on 5 March 2010, before rapid growth of the plants. Each treatment group had ten replicates. A total of 60 pots were established. All pots were measured gravimetrically by weighing and watered with distilled water every other day at 18:00 pm. On 25 March 2010, the drought treatments were initiated in half of the pots by withholding irrigation; the remaining pots continued to be well watered. The experimental treatments were conducted from 25 March to 15 June (when the plants were harvested), 2010.

### Photosynthetic parameters

On 24 April 2010, a cloudless day, the diurnal variation in the leaf net photosynthetic rate (Pn), stomatal conductance (Gs), inter-cellular CO_2_ concentration (Ci), transpiration rate (Tr), as well as ambient CO_2_ concentration (Ca), air temperature (Ta), air relative humidity (RH) and photosynthetically active radiation (PAR), were measured every two hours from 09:00 to 19:00 using a portable photosynthesis system (LI-6400, Li-Cor, Lincoln, NE, USA). The measurements were conducted on the second fully expanded leaves from 6 individual plants per treatment. The stomatal limitation value (Ls) was calculated using the formula: Ls = 1 – Ci/Ca [20]

### Chl fluorescence

Chl fluorescence was determined on fully expanded and exposed leaves (one leaf per plant) using a modulated fluorometer (PAM 2100, Walz, Effeltrich, Germany) on April 25 2014, according to [15]. Initial fluorescence (F0) and maximal fluorescence (Fm) were measured after a 30 min dark adaptation. The intensity of the saturation pulses used to determine the maximal fluorescence emission in the presence (Fm′) and absence (Fm) of quenching was 8000 μmol (photon) m^−2^ s^−1^, 0.8 s, whereas the “actinic light” was 336 μmol (photon) m^−2^ s^−1^. Steady-state fluorescence (Fs), basic fluorescence after light induction (F0′), maximal PS2 photochemical efficiency (Fv/Fm), effective quantum yield of PS2 (ФPS2), and photochemical (qP) and non-photochemical (qN) fluorescence quenching coefficients were also recorded. The effective quantum yield of photochemical energy conservation in PS2 (Fv′/Fm′) was calculated as (Fm′ − Fs)/Fm′ according to previous research [25].

### Determination of WUE and photosynthetic pigments

In this experiment under controlled conditions, WUE was studied at the level of the leaf instantaneous ratio of Pn to Tr (WUEi) [16]. After the determination of photosynthetic activity, all leaves were harvested. Fresh leaves (0.1 g) were collected for the determination of Chl content. The leaves were ground in 80 % acetone for the extraction of Chl and carotenoids (Car). The absorbance of the extract was measured at 645 and 663 nm using a UV/visible spectrophotometer (Lambda 25, Perkin Elmer, CT, USA).

### Statistical analysis

Significant differences between the water and fertilization treatments (n = 6) at a particular measurement point were analyzed with one-way ANOVA using SPSS 16.0 for Windows (Chicago, USA). The main effects of water and fertilizer availability and their interactions were determined using two-way analysis of variance (ANOVA). The differences were considered significant at *P* < 0.05.
